# Supplementary material for: Patterns of Intron Gain and Loss in Fungi
Source: PLoS Biol. 2004 Nov 30;2(12):e422. doi: 10.1371/journal.pbio.0020422 (PMC532390; doi:10.1371/journal.pbio.0020422)
Supplement: Table S1 — Also available at http://genes.mit.edu/NielsenEtAl/. (4.3 MB ZIP). [file pbio.0020422.st001.zip › NielsenEtAl/html/414.html]

AN4777.1.NCU00618.1.MG02872.1.FG06407.1


```
 CLUSTAL W (1.82) Multiple Sequence Alignments - Introns Inserted


Sequence 1: MG02872.1	82 aa
Sequence 2: FG06407.1	75 aa
Sequence 3: NCU00618.1	82 aa
Sequence 4: AN4777.1	82 aa
Alignment Length: 82 aa
Number Identitical Residues: 64 aa
Alignment Score (without introns) 2856


MG02872.1 	M0VLAVDLLNPSPAAEAKKHKLK0TLVPAPRSFFMDVKCPGCFTITTVFSHAQTVVICQG
NCU00618.1	M0VLAVDLLNPSPASEARKHKLK0TLVPAPRSFFMDVKCPGCFTITTVFSHAQTVVICQG
FG06407.1 	-~------MNPSPAAEAKKHKLK0TLVPAPRSFFMDVKCPGCFTITTVFSHAQTVVICQG
AN4777.1  	M0VLAVDLLNPTPQAEARKHKLK0QLVPGPRSFFMDVKCPGCFNITTVFSHAQTVVVCSG
          	    : . :**:* :**:*****  ***.**************.************:*.*

MG02872.1 	CTTVLCQPTGGKARLTEGCSFRRK
NCU00618.1	CTTVLCQPTGGKARLTEGCSFRRK
FG06407.1 	CTTVLCQPTGGKARLTEGCSFRRK
AN4777.1  	CSTVLCQPTGGKARLTEGCSFRRK
          	*:**********************
```
